# Reversible superconductor-insulator transition in LiTi_2_O_4_ induced by Li-ion electrochemical reaction

**DOI:** 10.1038/srep16325

**Published:** 2015-11-06

**Authors:** K. Yoshimatsu, M. Niwa, H. Mashiko, T. Oshima, A. Ohtomo

**Affiliations:** 1 Department of Applied Chemistry, Tokyo Institute of Technology, 2-12-1 Ookayama, Meguro-ku, Tokyo 152-8552, Japan; 2 Materials Research Centre for Element Strategy (MCES), Tokyo Institute of Technology, Yokohama 226-8503, Japan

## Abstract

Transition metal oxides display various electronic and magnetic phases such as high-temperature superconductivity. Controlling such exotic properties by applying an external field is one of the biggest continuous challenges in condensed matter physics. Here, we demonstrate clear superconductor-insulator transition of LiTi_2_O_4_ films induced by Li-ion electrochemical reaction. A compact electrochemical cell of pseudo-Li-ion battery structure is formed with a superconducting LiTi_2_O_4_ film as an anode. Li content in the film is controlled by applying a constant redox voltage. An insulating state is achieved by Li-ion intercalation to the superconducting film by applying reduction potential. In contrast, the superconducting state is reproduced by applying oxidation potential to the Li-ion intercalated film. Moreover, superconducting transition temperature is also recovered after a number of cycles of Li-ion electrochemical reactions. This complete reversible transition originates in difference in potentials required for deintercalation of initially contained and electrochemically intercalated Li^+^ ions.

Development of rechargeable Li-ion batteries with high cell-potential and capacity has become increasingly important for sustainable society^1^. Electrochemical reactions with Li^+^ ions involve intercalation to the anode and cathode materials, corresponding to charge and discharge of the batteries. Such a Li-ion intercalation is also utilized for modulation of materials properties; creation of a new superconductor that cannot be synthesized by conventional solid-state reactions. Various superconductors have been synthesized by the electrochemical Li-ion intercalation, *e.g.* Li_*x*_*M*NCl (*M* = Zr and Hf), Li_*x*_Sr_2_CuO_2_ X_2_ (X = Cl, Br, and I), Li_*x*_*A*Sr_2_Nb_3_O_10_ (*A* = Rb and Cs) and related nonstoichiometric compounds^2^,^3^,^4^,^5^. The most electropositive Li^+^ ions enable to dope a large number of electrons in parent materials although a range of the doping is limited to space available for intercalation of excess Li^+^ ions. The degree of nonstoichiometry is also important for battery operation to keep high capacity without degradation of the electrode materials.

Amongst a variety of materials, LiTi_2_O_4_ (LTO) is a unique candidate for our experiment with the use of a Li-ion battery structure because LTO exhibits superconductivity with a critical temperature as high as 13.7 K^6^,^7^,^8^,^9^,^10^,^11^,^12^,^13^,^14^,^15^. LTO has a spinel-type structure with a lattice constant of *a* = 8.40 Å^6^. Li^+^ ions are located at the centre of oxygen tetrahedrons and Ti^3+^ and Ti^4+^ ions are of oxygen octahedrons^6^,^7^,^8^,^9^,^10^. The edge-shared TiO_6_ octahedrons form a conduction path of electrons and the LiO_4_ tetrahedrons play a role of charge reservoir to accommodate Ti sites with the carriers. The occupancy of Li^+^ ions at the tetrahedral sites is one-fourth for the ideal chemical formula. The excess Li^+^ ions can be introduced into empty space either at tetrahedral and octahedral sites. This situation is regarded as *over-doping*. Meanwhile, the excess Li^+^ ions also replace the Ti ions resulting in hole doping in addition to interruption of the edge-shared TiO_6_ network. Therefore, the electronic property of LTO dramatically changes depending on content and location of Li^+^ ions. In particular, superconductivity disappears and then metallic phase eventually undergoes to a transition to insulator with either increasing or decreasing Li content from the ideal chemical formula^8^,^9^,^10^. These facts suggest that superconductivity can be modulated by Li-ion electrochemical reactions.

Hamada *et al.* reported Li-ion electrochemical reaction to bulk LTO^11^. Li content *x* of bulk Li_1+*x*_Ti_2_O_4_ was controlled in a range of –0.3 ≤ *x* ≤ 1 and found that superconducting transition temperature (*T*_C_) of LTO varied in a range from ~12 to 13.3 K. Superconductor-insulator transition (SIT) was not observed due to phase separation, which would be caused by inhomogeneous electrochemical reaction of bulk LTO with larger volume and smaller surface area.

In this article, we demonstrate clear SIT of LTO films induced by Li-ion electrochemical reaction. A compact electrochemical cell of pseudo-Li-ion battery structure was formed with a superconducting LTO film as an anode^16^. By applying a constant voltage exceeding reduction potential, Li content in the LTO film increased and the sample showed insulating behaviour. In contrast, by applying a constant voltage over oxidation potential, electrochemical deintercalation of Li^+^ ions occurred to regain superconductivity. In addition, *T*_C_ was found to be almost identical even after a number of repeats for Li-ion intercalation/deintercalation reactions. The fact that after the repeated experiments the crystal structure of LTO film remained intact manifested itself in the complete and reversible SIT.

## Results

### Li-ion electrochemical reaction

**Figure 1:**
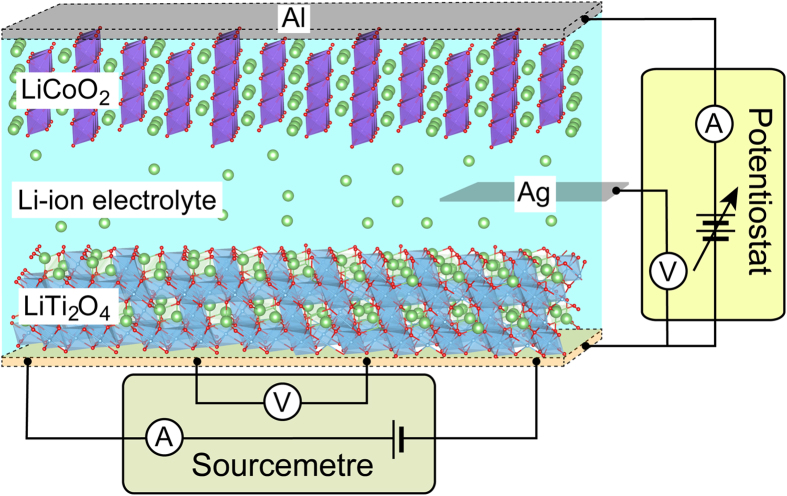
Schematic illustration of the pseudo-Li-ion battery structure. The anode and cathode materials are LiTi_2_O_4_ film and LiCoO_2_/Al, respectively. The Li-ion electrochemical reactions and resistivity measurements are independently performed by using electric circuits, drawn by at the side and bottom of the cell, respectively.

Our setup as illustrated in Fig. 1, Li-ion electrolyte sandwiched by LiCoO_2_/Al foil (cathode) and LTO film (anode), can be regarded as pseudo-Li-ion battery structure. Four-probe resistivity measurements and three-terminal Li-ion electrochemical reactions were performed by using a sourcemetre and a potentiostat, respectively. When a potential lower than the Ti^3+^/Ti^4+^ redox potential is applied, Li^+^ ions are intercalated into the LTO film, being analogous to charging battery. To balance the positive charge of the Li^+^ ions, electrons are injected into the film, resulting in the reduction of the Ti valence. Thus, application of negative potential to the anode corresponds to effective electron doping owing to the most electropositive ionicity of Li. In contrast, application of positive potential results in Li-ion deintercalation (removal of Li^+^ ions from the *over-doped* film), which corresponds to hole doping and thus discharging battery.

**Figure 2:**
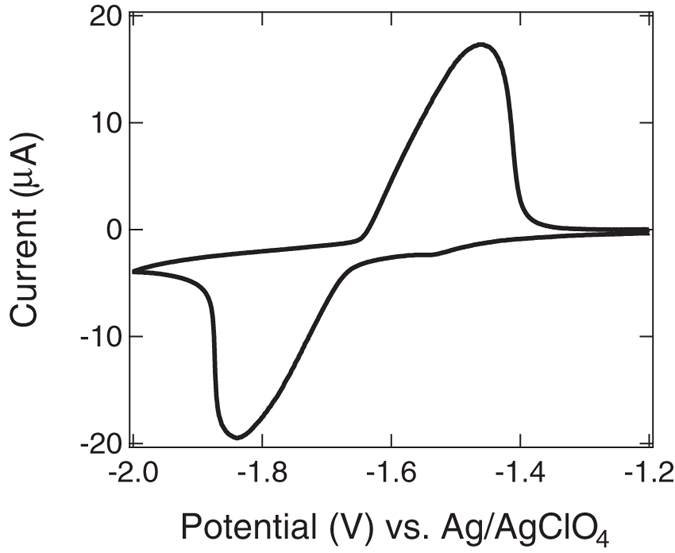
Li-ion electrochemical reaction of LiTi_2_O_4_ films. Cyclic voltammogram of LiTi_2_O_4_|LiClO_4_:PC|LiCoO_2_/Al system at a rate of 5 mV/s.

We first studied aforementioned Li-ion electrochemical reactions by cyclic voltammetry. Figure 2 shows typical cyclic voltammogram of LTO|LiClO_4_:propylene carbonate (PC)| LiCoO_2_/Al system taken at a rate of 5 mV/s. Rotationally symmetric curves with equal peak integral were clearly observed. The cathodic peak at −1.8 V (vs. Ag/AgClO_4_) was attributed to the reduction of Ti^4+^ to Ti^3+^, whereas subsequent oxidation of Ti^3+^ to Ti^4+^ represented the anodic peak at −1.5 V. We also measured cyclic voltammogram in a wide potential range from −4 to +1.5 V (vs. Ag/AgClO_4_). Decomposition of LTO films occurred at around +0.3 V, and deposition of Li metal at the surface of the films occurred below −3 V (see Supplementary Note 1 online). From these characteristic cyclic voltammograms, the applied potentials in the chronoamperometric Li-ion intercalation (deintercalation) were set at −2.0 (−0.5) V to guarantee reversible Li-ion electrochemical reactions and stable battery operation.

### Structural characterization

**Figure 3:**
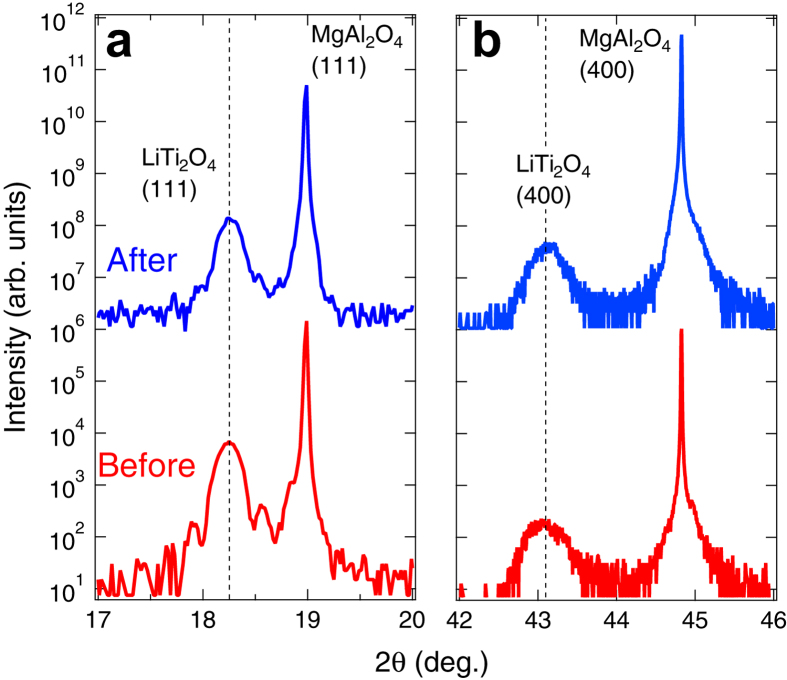
Structural characterization of LiTi_2_O_4_ films. XRD patterns for the LiTi_2_O_4_ films before (red) and after (blue) Li-ion electrochemical reactions. (**a**) XRD pattern around the symmetric (111) MgAl_2_O_4_ reflection. (**b**) XRD pattern around the asymmetric (400) MgAl_2_O_4_ reflection.

**Figure 4:**
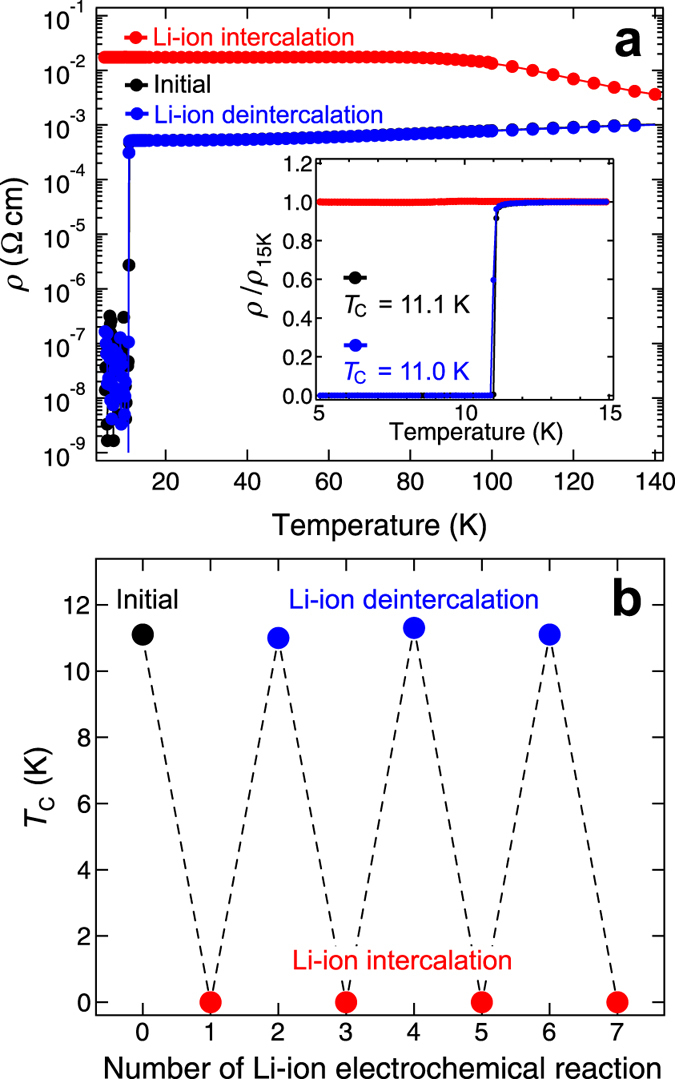
Reversible superconductor-to-insulator transition of LiTi_2_O_4_ films induced by Li-ion electrochemical reactions. (**a**) The black, red, and blue markers indicate temperature-dependent resistivity for initial, intercalated (1st), and deintercalated (1st) LiTi_2_O_4_ films, respectively. The inset shows the magnified curves near *T*_C_ (normalized by the values at 15 K). Note that the initial and the deintercalated curves are overlapped to each other. (**b**) Passage of *T*_C_ against the number of repeats of Li-ion electrochemical reactions. The initial data is set at 0. For convenience, *T*_C_ of 0 K for all the Li-ion intercalated films represents the fact that resistivity did not fall to zero above 2 K.

The chemical and structural stability of the LTO anode was also confirmed by x-ray diffraction (XRD). Figure 3(a,b) show the symmetric and asymmetric XRD patterns around LTO (111) and (400) reflections for the samples before and after Li-ion electrochemical reactions, respectively. We noted that the data shown in Fig. 3(a,b) were taken from the film with which the temperature dependence of resistivity shown in Fig. 4(a) was measured. The (111) reflection of the as-grown LTO film was detected at 2θ = 18.25°, corresponding to *d*_111_ = 4.86 Å. The film after the reaction exhibited this reflection at the exactly same angle. In addition, the asymmetric (400) reflections of LTO film were also detected 2θ = 43.1°, corresponding to *d*_400_ = 2.10 Å, regardless of Li-ion electrochemical reactions. The calculated lattice constant of the LTO films (*a* = 8.40 Å) agreed with that of bulk^7^,^9^. We also performed wide-range out-of-plane XRD measurements for both samples to find possible segregation of secondary phase (see Supplementary Note 2 online). Such a trace was not detected, indicating that the crystal structure remained intact during Li-ion electrochemical reactions^17^,^18^. We also investigated surface morphology of the LTO films before and after Li-ion electrochemical reactions by using atomic force microscopy (AFM) (see Supplementary Note 3 online). Both samples indicated flat surface with a root mean square roughness of less than 0.5 nm, but not a sign of corrosion such as dissolution, segregation, and deposition. These data suggest that all of the following results have been established in the LTO film of original quality that remains intact during reversible Li-ion intercalation/deintercalation.

### Superconductor-insulator transition

Electrochemical modulation of superconducting properties was investigated by measuring temperature dependence of resistivity. The initial LTO film (immersed in electrolyte) showed metallic conductivity with room-temperature resistivity of ~1 × 10^−3^ Ω cm and a superconducting transition at *T*_C_ = 11.1 K [Fig. 4(a)]. We noted that lower *T*_C_ of our LTO film than that of bulk was caused by slight nonstoichiometry of LTO films. It was difficult to make Li content exactly unity because of high vapour pressure of Li. (see ref. 15 for details) In contrast, the Li-ion intercalated film showed insulating behaviour with resistivity higher than the initial value by an order of magnitude. Moreover, the resistivity increased with decreasing temperature and eventually became a constant at temperatures from 80 to 2 K. The absence of the superconducting state indicates occurrence of SIT. We continued measurements after Li-ion deintercalation to find that resistivity curve was in excellent agreement with the initial one, including *T*_C_ [see inset of Fig. 4(a)]. These results indicate that the superconducting state was completely regained after a cycle of electrochemical reaction.

Excellent reversibility in SIT was further seen in *T*_C_ as a function of number of the Li-ion electrochemical reactions [Fig. 4(b)]. *T*_C_ was nearly constant even after three cycles (deviation in *T*_C_ was less than 0.3 K). Further measurement was only hampered by slow evaporation of Li-ion electrolyte. Except for this drawback, our setup including the use of thin film with large surface area is ideal for electrochemical modulation of the electronic state in LTO.

## Discussion

Now, we would like to discuss possible mechanism of SIT in the LTO film. Taking strong dependence of *T*_C_ on Li content for bulk LTO into account, nearly constant *T*_C_ in repeated experiments suggests that the Li contents in the initial and Li-ion deintercalated films are almost identical to each other, which implies that Li^+^ ions contained in the as-grown film were immobile and did not contribute electrochemical reactions. In fact, when the Li-ion *deintercalation* was first applied to another LTO film, its superconducting state remained although *T*_C_ slightly changed (see Supplementary Note 4 online). The following Li-ion intercalation/deintercalation reactions (*i.e.* stepwise introduction/removal of excess Li^+^ ions) did not influence further change in *T*_*C*_. These results indicate that the chronoamperometric Li-ion intercalation (−2.0 V) and deintercalation (−0.5 V) induce simple transfer of a part of excess Li^+^ ions between the LTO film and Li-ion electrolyte.

This scenario does not necessarily interpret that the Li^+^ ions cannot be removed from an initial film by any Li-ion electrochemical reactions. When *chronopotentiometric* Li-ion deintercalation was applied to the initial LTO film, the oxidation current was clearly observed. (see Supplementary Note 5 online). Meanwhile, potential was found to be about −0.2 V (vs. Ag/AgClO_4_), which was higher than that applied in the chronoamperometric Li-ion deintercalation. The potential of −0.5 V (vs. Ag/AgClO_4_) was just too low to deintercalate the initially contained Li ions. Therefore, only the electrochemically intercalated Li^+^ ions could be deintercalated in the chronoamperometric Li-ion deintercalation, resulting in demonstration of complete reversible SIT of LTO film. Finally, we would like to emphasize that chronopotentiometric Li-ion electrochemical reactions are advantageous to subtle tuning of Li content in the films with monitoring redox currents. Using this method, the superconducting states with intermediate *T*_C_ were achieved (see Supplementary Note 6 online).

In summary, we have demonstrated SIT of LTO films induced by Li-ion electrochemical reactions. Li-ion intercalation to the LTO film can control the electronic phase from superconductor to insulator. The intercalated Li^+^ ions can be completely removed by applying oxidation potential, resulting in reproduction of the superconducting state. In addition, *T*_*C*_ is also recovered after a number of cycles of Li-ion electrochemical reactions. This complete reversible SIT originates in difference in potentials required for deintercalation of initially contained and electrochemically intercalated Li^+^ ions. Our study will pave a way to control electronic phase of thin layers electrochemically and *in-situ* and thus to reveal a complete electronic phase diagram in temperature and phase space.

## Methods

### Thin-Film Preparation

LTO films with thickness of approximately 50 nm were grown on (111) MgAl_2_O_4_ substrates by using pulsed-laser deposition technique in an ultrahigh-vacuum chamber. KrF excimer laser pulses (10 Hz, 0.7 J/cm^2^) were focused on a Li-excess ceramic tablet (LiTiO_x_). The Li-excess target was necessary for growing the high-quality LTO films because of significant loss of Li species during the ablation^13^,^15^. Substrate temperature was set 750 °C and Ar gas (6 N purity) was continuously fed into the camber with keeping the pressure of 0.1 mTorr during the growth. After the growth, the LTO films were cooled to room temperature under an Ar atmosphere.

### Electrochemical Reactions and Resistivity Measurements

Li-ion electrochemical reactions were performed with using the standard three-electrode setup. The working, counter, and reference electrodes consisted of LTO films, LiCoO_2_ coated Al foil, and Ag wire, respectively. Liquid electrolyte was prepared by mixing lithium ion salt of LiClO_4_ (>99%) and organic solvent of PC (>98%) (weight ratio of LiClO_4_ to PC = 1:20) in a glovebox, followed by heating at ~50 °C to remove moisture. Cyclic voltammetry was performed with using a potentiostat (model 2323, ALS Co., Ltd). The Li-ion electrochemical reactions and successive temperature-dependent resistivity measurements were performed *in-situ* by using a four-probe method using physical properties measurement system (Quantum design, PPMS). This setup prevented the sample and Li-ion electrolyte from degradation caused by exposure to air. For four-probe resistivity measurements, Al wire (25 μm diametre) was directly welded to the surface of LTO films.

One cycle of the experiment was started from electrochemical Li-ion intercalation (deintercalation) to the LTO film performed at room temperature by applying a potential of −2.0 V (−0.5 V) with respect to the Ag/AgClO_4_ quasi-reference electrode. While keeping constant potential, the cell was cooled to 140 K at a rate of 10 K/min, with applying the continuous constant potential in order to prevent self-discharge. At 140 K, motion of the Li^+^ ions was completely frozen in a solidified medium, so that we stopped applying external potential and run resistivity measurements. After temperature reached 2 K, the cell was warmed to room temperature at a rate of 10 K/min.

### Structural Characterizations

Before and after Li-ion electrochemical reactions, crystal structures and surface morphology of the LTO films were inspected by a Cu Kα_1_ XRD apparatus and AFM, respectively. Here, the Li-ion electrochemical reaction corresponds to applying a reduction potential of –2.0 V (vs. Ag/AgClO_4_) to the LTO film. Then, the film was washed with deionized water in an ultrasonic cleaner and dried by blowing N_2_ gas.

## Additional Information

**How to cite this article**: Yoshimatsu, K. *et al.* Reversible superconductor-insulator transition in LiTi_2_O_4_ induced by Li-ion electrochemical reaction. *Sci. Rep.* **5**, 16325; doi: 10.1038/srep16325 (2015).

## Supplementary Material

Supplementary Information
