# Supplementary material for: Reversible superconductor-insulator transition in LiTi2O4 induced by Li-ion electrochemical reaction
Source: Sci Rep. 2015 Nov 6;5:16325. doi: 10.1038/srep16325 (PMC4635382; doi:10.1038/srep16325)
Supplement: Supplementary Information [file srep16325-s1.pdf]

Supplementary Information

Reversible superconductor-insulator transition in $LiTi_2O_4$ induced by Li-ion electrochemical reaction

K. Yoshimatsu[1,*], M. Niwa[1], H. Mashiko[1], T. Oshima[1] & A. Ohtomo[1,2]

[1]Department of Applied Chemistry, Tokyo Institute of Technology, 2-12-1 Ookayama, Meguro-ku, Tokyo 152-8552, Japan.
[2]Materials Research Centre for Element Strategy (MCES), Tokyo Institute of Technology, Yokohama 226-8503, Japan.
Correspondence and requests for materials should be addressed to K.Y. (email: k-yoshi@apc.titech.ac.jp)

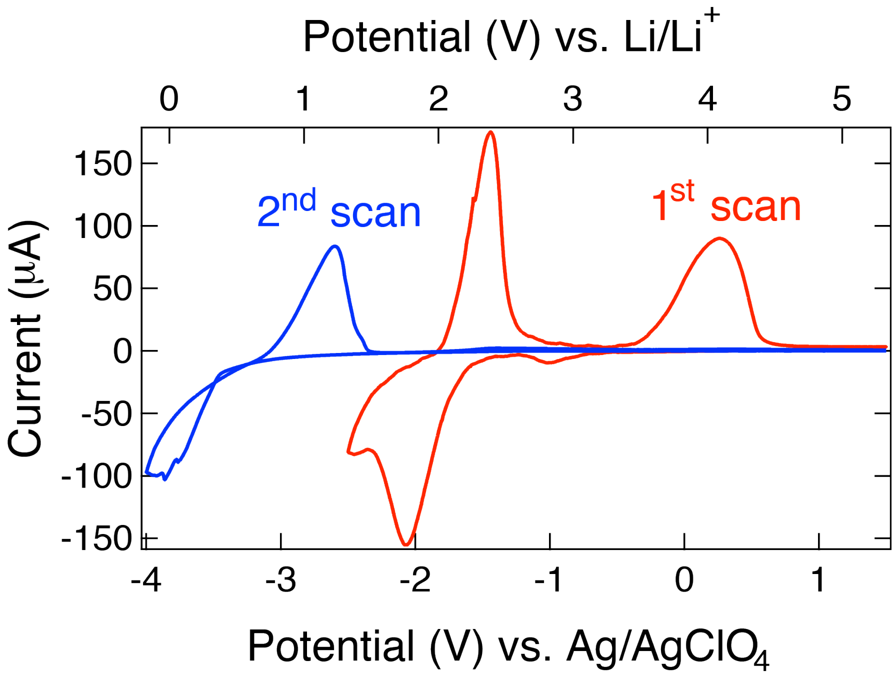

Figure S1. Wide-potential range cyclic voltammograms of $LiTi_2O_4$|$LiClO_4$:propylene carbonate|Pt system. The red and blue colours indicate the 1st and 2nd voltage sweeps, respectively.

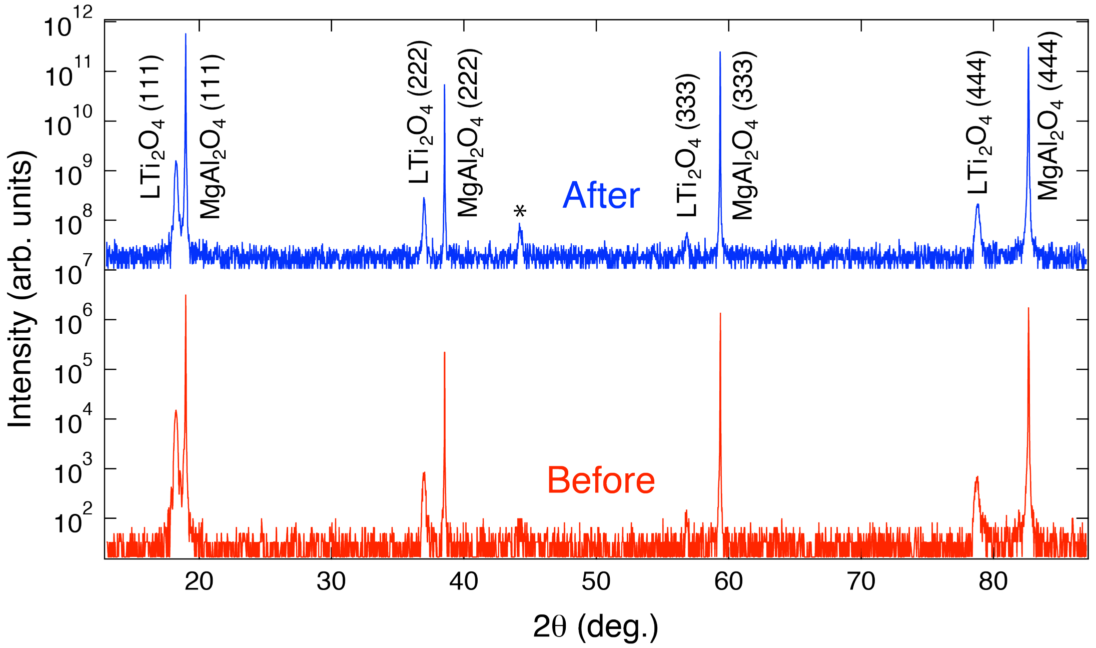

Figure S2. Wide-range out-of-plane x-ray diffraction patterns of $LiTi_2O_4$ films before and after Li-ion electrochemical reactions. The asterisk indicates a reflection coming from the sample stage.

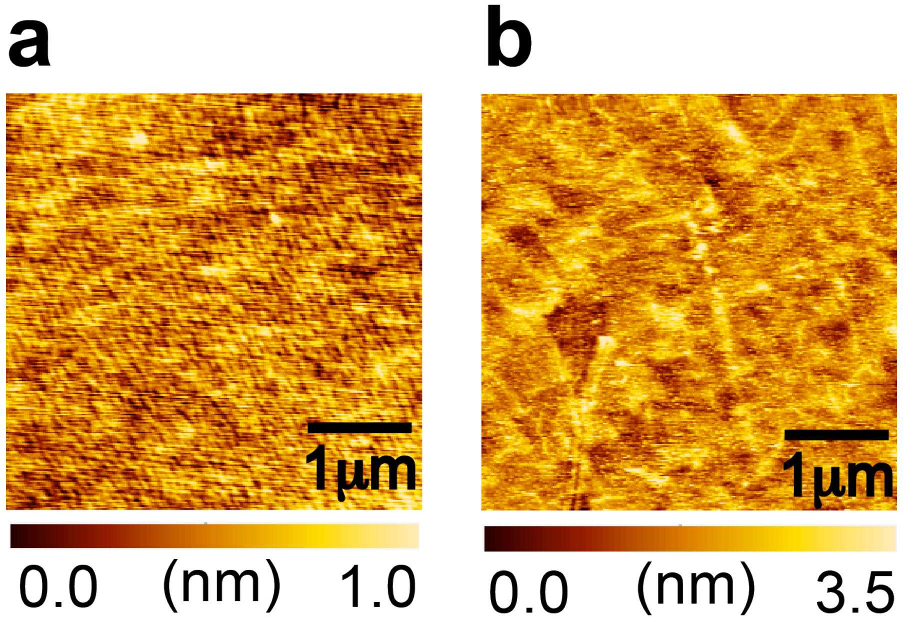

Figure S3. Atomic force microscopy images of the $LiTi_2O_4$ films (**a**) before and (**b**) after Li-ion electrochemical reactions.

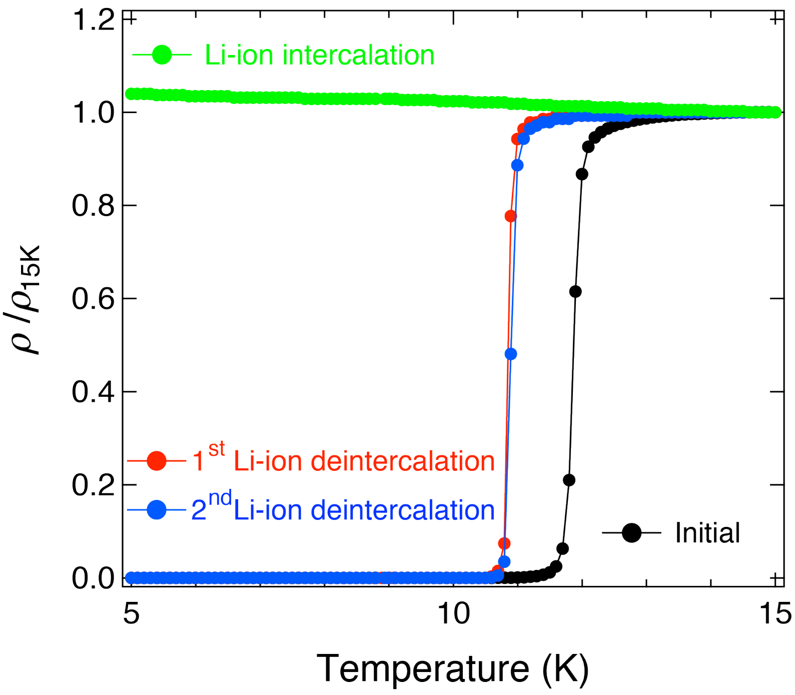

Figure S4. Temperature dependence of normalized resistivity for $LiTi_2O_4$ films. The black, red, green, and blue colours indicate the initial, 1st Li-ion deintercalated, Li-ion intercalated, and 2nd Li-ion deintercalated $LiTi_2O_4$ films, respectively.

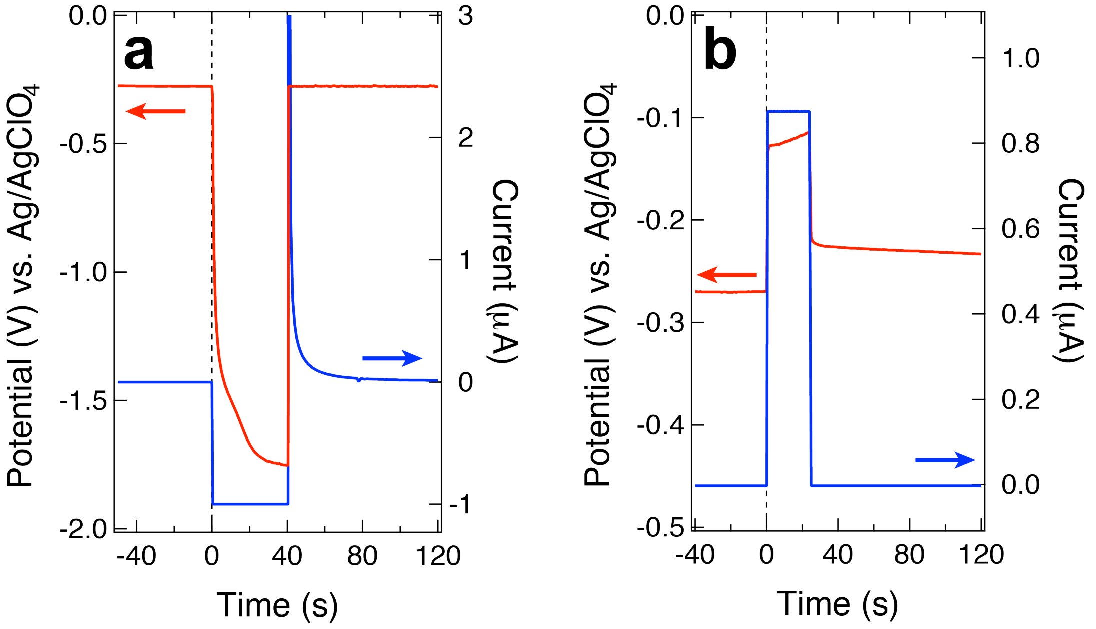

Figure S5. Plots of redox current and potential during (**a**) Li-ion intercalation and (**b**) Li-ion deintercalation reactions to the initial $LiTi_2O_4$ film.

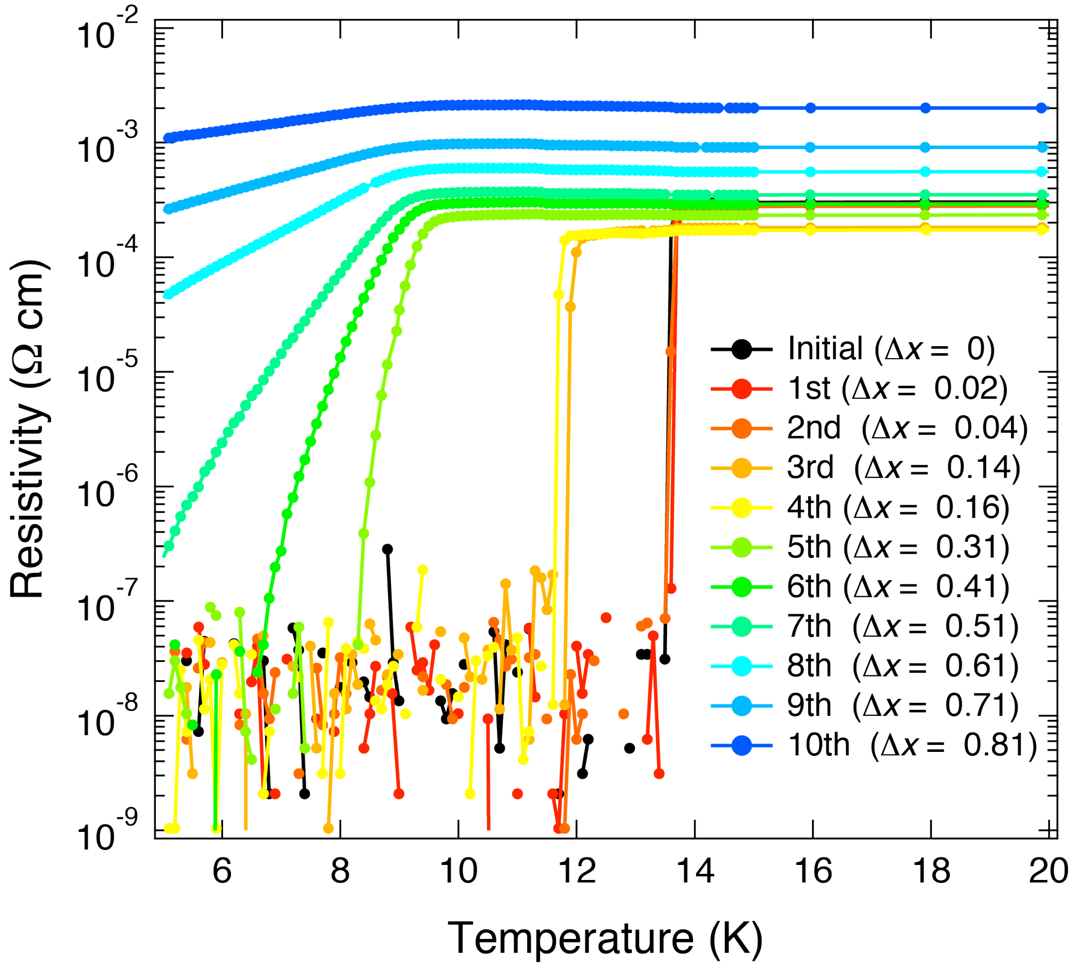

Figure S6. Temperature dependence of resistivity for $LiTi_2O_4$ films with various Li content. Δx represents the variation of Li content in $LiTi_2O_4$ chemical formula.

Supplementary Note 1:

Cyclic voltammetry for $LiTi_2O_4|LiClO_4$:PC|Pt system

Figure S1 shows cyclic voltammograms measured in the extended potential range. The 1st scan was taken in a range from −2.5 V to +1.5 V (vs. Ag/$AgClO_4$ quasi-reference electrode). In addition to peaks of a Ti^{3+}/Ti^{4+} redox couple, a characteristic anodic peak was found at +0.2 V. In the 2nd scan (from −4.0 V to +1.5 V), all of these peaks were absent, while a new redox couple appeared, which was associated with deposition and dissolution of Li in electrolyte. Therefore, the peak at +0.5 V, only visible in the 1st scan can be attributed to decomposition and/or delamination of the $LiTi_2O_4$ film. These results suggest that in a potential range of −2 V to −0.5 V reversible reactions of the Ti^{3+}/Ti^{4+} redox couple in $LiTi_2O_4$ films occur and other components of the system can be kept electrochemically inactive.

Supplementary Note 2:

Crystal structure of $LiTi_2O_4$ films

Figure S2 shows wide-range out-of-plane x-ray diffraction (XRD) patterns for the $LiTi_2O_4$ films before and after Li-ion electrochemical reactions. The (*hhh*) reflections (h = 1, 2, 3, and 4) of $MgAl_2O_4$ substrates and $LiTi_2O_4$ films were detected in the XRD patterns. Note that the small peak at $2\theta \sim 45^o$ (indicated by the asterisk) is reflection coming from the sample stage (size of the samples used for the pseudo-Li-ion battery structure was smaller than spot area of incident x-ray). No other peaks were found in the XRD patterns.

Supplementary Note 3:

Surface morphology of $LiTi_2O_4$ films

Surface morphology was investigated by atomic force microscopy (AFM). Figure S3(a) and S3(b) show AFM images of the $LiTi_2O_4$ films before and after Li-ion electrochemical reactions, respectively. Both images show flat surfaces with a root mean square roughness of less than 0.5 nm. As for Fig. S3(b) thin and shallow crevices are visible, which are presumably due to trace of some grain boundaries being prominent during the repeated temperature variation for a series of resistivity measurements. Otherwise, any sign of dissolution and/or deposition of $LiTi_2O_4$ were not observed in the images.

Supplementary Note 4:

Chronoamperometric Li-ion deintercalation to the initial $LiTi_2O_4$ films

Figure S4 shows temperature dependence of resistivity of initial, Li-ion deintercalated, and Li-ion intercalated $LiTi_2O_4$ films. The resistivity curves were normalized by the values 15 K. The initial $LiTi_2O_4$ film, which is a different sample from one described in the main text, showed superconductivity below $T_C = 11.9$ K. The oxidation potential [–0.5 V (vs. Ag/$AgClO_4$)] was applied to the initial $LiTi_2O_4$ films (referred as to the 1st Li-ion deintercalation). Then, the film also showed superconductivity although T_C slightly decreased to 11.0 K. In contrast, when the reduction potential [–2.0 V (vs. Ag/$AgClO_4$)] was applied to the 1st Li-ion deintercalated film, it became an insulator above 2K. By carrying out next the Li-ion deintercalation reaction to the Li-ion intercalated $LiTi_2O_4$ film (referred as to the 2nd Li-ion deintercalation), it again became a superconductor and T_C coincide with that of the 1st one. These results demonstrate reversible SIT, being in good agreement with data described in the main text (Fig. 4). Moreover, the results indicate that order of Li-ion intercalation and deintercalation reactions does not influence on the superconductor-insulator transition at all. The insulating states are realised only when the reduction potential is applied to the $LiTi_2O_4$ film, regardless of history of potential variation.

Supplementary Note 5:

Chronopotentiometric Li-ion intercalation/deintercalation to the $LiTi_2O_4$ film

Figure S5(a) and S5(b) show plots of redox current and potential measured during chronopotentiometric Li-ion intercalation and deintercalation to the initial $LiTi_2O_4$ films, respectively. The open circuit potential at zero current was found to be –0.3 V (vs. $Ag/AgClO_4$). During the Li-ion intercalation reaction, the reduction current was ~1.0 μA and the potential became lower as the reaction proceeded. When 40 s passed after starting Li-ion intercalation to the film, potential dropped to –1.8 V [Fig. S5(a)]. This potential is higher than that applied in the chronoamperometric Li-ion intercalation, indicating that Li ions were indeed intercalated into the initial $LiTi_2O_4$ film by applying constant potential of –2.0 V.

In contrast, chronopotentiometric Li-ion deintercalation revealed that the potential of –0.5 V is too low to remove Li ions from the initial $LiTi_2O_4$ film. As shown in Fig. S5(b), during the chronopotentiometric Li-ion deintercalation, the potential varied from –0.3 to –0.2 V as the Li-ion deintercalation proceeded. This potential range is higher than the applied potential during the chronoamperometric Li-ion deintercalation, indicating that Li ions were not removed from the initial $LiTi_2O_4$ film by applying the constant potential of –0.5 V.

Supplementary Note 6:

The observation of intermediate T_C for $LiTi_2O_4$ films subjected to chronopotentiometric Li-ion intercalations.

Figure S6 shows temperature dependence of resistivity for $LiTi_2O_4$ films with various Li content. Chronopotentiometric Li-ion intercalation reactions were performed to control a small number of Li ions. Here, Δx (increment of Li content in $LiTi_2O_4$ chemical formula) was estimated from the reduction currents during the Li-ion intercalation reactions. Systematic variation of T_C as well as resistivity at normal states was clearly observed. Note that $T_{C,onset}$ of 13.7 and 9.1 K (estimated for samples with Δx = 0.02 and 0.41, respectively) indicates a range of successive modulation within a superconducting phase. In contrast, when Δx exceeded 0.41, the SIT became broader and zero resistivity was not observed above 5K. These results suggested that the Li content of the $LiTi_2O_4$ Δx = 0.41 film was close to the upper critical limit of Li (electron) doping in the superconducting phase.
